# Microbiome-metabolomics analysis provides insight into the effects of dietary taurine on meat quality and flavor in yellow-feathered chickens (*Gallus gallus*)

**DOI:** 10.1016/j.aninu.2026.04.013

**Published:** 2026-09-07

**Authors:** Yujie Huang, Ahmed Mohamed Fouad, Jinling Ye, HebatAllah Kasem El-Senousey, Xiajing Lin, Sai Zhang, Yongwen Zhu, Shijuan Yan, Zongyong Jiang, Shouqun Jiang, Dong Ruan

**Affiliations:** a State Key Laboratory of Swine and Poultry Breeding Industry, Key Laboratory of Animal Nutrition and Feed Science in South China, Ministry of Agriculture and Rural Affairs, Guangdong Provincial Key Laboratory of Animal Breeding and Nutrition, Institute of Animal Science, Guangdong Academy of Agricultural Sciences, Guangzhou 510640, Guangdong, China; b Guangdong Provincial Key Laboratory of Animal Nutrition and Regulation, College of Animal Science, South China Agricultural University, Guangzhou 510642, Guangdong, China; c Department of Animal Production, Faculty of Agriculture, Cairo University, Giza 12613, Egypt; d Guangdong Key Laboratory for Crop Germplasm Resources Preservation and Utilization, Agro-biological Gene Research Center, Guangdong Academy of Agricultural Sciences, Guangzhou 510640, Guangdong, China

**Keywords:** Taurine, Meat quality and flavor, Gut microbiome, Lipidome, Broiler chicken

## Abstract

Taurine (TAU) is a crucial component in poultry nutrition, recognized for its potential to improve broiler growth and enhance meat quality and flavor. However, the mechanisms by which TAU regulates the production of volatile organic compounds (VOC) in broiler meat remain unknown. This study investigated the effects of TAU supplementation on the meat quality, lipid metabolism-related gene expression, and the VOC profile in yellow-feathered chickens. Nine hundred healthy 1-d-old female fast-growing *Lingnan* yellow-feathered chickens (body weight: 38.58 ± 0.12 g) were randomly assigned to 5 groups, and were fed either a basal diet (CON) or a basal diet supplemented with 0.25, 0.50, 1.00, and 2.00 g/kg TAU (TAU250, TAU500, TAU1000, and TAU2000, respectively) for 56 d. The findings revealed that dietary TAU significantly increased the ADG and final BW, while decreased F/G during d 1 to 56 (*P* < 0.05), compared with the CON group. Dietary supplementation with 2.0 g/kg TAU increased C18:1, C18:2n-6, and redness (a∗) value at 45 min (*P* < 0.05), while decreasing 24-h drip loss in breast muscle compared with the CON group (*P* < 0.001). Dietary TAU supplementation significantly increased the contents of triglycerides, phosphatidylethanolamines, phosphatidylcholines, phosphatidylinositols, and lysophosphatidylcholines lipid subclasses (*P* < 0.05). The TAU enhanced intramuscular fat deposition by downregulating lipid catabolism-related genes, including *CPT1A*, *ACOX1*, and *ACSL5* in breast muscle (*P* < 0.05). Moreover, dietary supplementation with 2.00 g/kg TAU increased *Bacteroides*, *Faecalibacterium* and *Enterococcus* and decreased *Streptococcus* and *Lachnoclostridium* (*P* < 0.05). Integrated analysis of flavoromics and gut microbiome profiling revealed that *Faecalibacterium* and *Enterococcus* were positively correlated with (E)-2-octenal, ethyl elaidate, ethyl myristate, ethyl palmitate, ethyl 9,12-octadecadienoic acid, and octane (*P* < 0.05). In conclusion, this study found that dietary TAU improved intramuscular fat deposition and promoted the synthesis of VOC in yellow-feathered chickens. *Faecalibacterium* and *Enterococcus* may help to improve the flavor profile of the meat.

## 1 Introduction

With the rapid development of the domestic economy, consumers are increasingly paying attention to meat quality attributes, such as freshness, tenderness and flavor. Yellow-feathered broiler chickens, unlike western white-feathered broilers, are very popular in Asia due to their superior meat quality (Li et al., 2020). Modern intensive broiler farming is challenged by oxidative stress, lipid deposition, and various diseases, which lead to impaired growth performance, reduced meat quality and limited accumulation of flavor precursors (Kong et al., 2023; Petracci et al., 2019).

Taurine (TAU), 2-aminoethanesulfonic acid, a non-protein sulfur β-amino acid, is an important component in poultry nutrition, particularly in plant-based poultry diets (He et al., 2021). Previous studies have revealed that TAU possesses strong antioxidant, anti-inflammatory, anti-apoptotic, and cytoprotective properties (Niu et al., 2018; Surai et al., 2021; Uyanga et al., 2022). Taurine regulates cellular energy metabolism, mitochondrial homeostasis, and promotes muscle cell development (Seidel et al., 2019). Taurine has been shown to inhibit lipid deposition and improve lipid metabolism (Kp and Martin, 2023). Additionally, it serves as an energy source for gut microbes and has the potential to alter microbiota composition (Singh et al., 2023). Such microbial restructuring can enhance host nutrient utilization, overall health and growth performance of poultry (Yadav and Jha, 2019). Among amino acids, TAU level was reported to be higher in muscles of Chinese local breeds compared with Western commercial strains (Chen et al., 2023). This elevated endogenous TAU level in local breeds may be associated with their distinct muscle fiber composition and slower growth rate. It has been reported that dietary TAU supplementation improved growth performance, meat quality, and the antioxidant capacity of muscle in Arbor Acres broilers, which is mechanistically linked to TAU's ability to suppress lipid oxidation and activate the nuclear factor erythroid 2-related factor 2 (Nrf2) antioxidant pathway, collectively enhancing meat water-holding capacity and tenderness (Han et al., 2020; Huang et al., 2014; Lu et al., 2020). However, the impacts of dietary TAU supplementation in yellow-feathered chickens have not been investigated yet. The flesh color, juiciness, flavor, and tenderness are important indices to measure chicken meat quality and also the main factors to determine product value. Volatile organic compounds (VOC) are important substances that affect meat flavor. Fatty acids influence the VOC profile of chicken by undergoing lipid oxidation and other chemical reactions, serving as precursors to flavor compounds (Mancinelli et al., 2021). In addition, certain bacteria that regulate lipid metabolism have been shown to increase muscle flavor precursors thereby influencing meat flavor (Liu et al., 2023a, Liu et al., 2023b; Ruan et al., 2024; Xu et al., 2024). Studies have shown that TAU regulates intestinal microflora and improves gut microenvironment (Qian et al., 2023; Yu et al., 2016).

However, how TAU can regulate the production of VOC remains largely unknown. To address this knowledge gap, this study investigated the impacts of dietary TAU supplementation on growth performance, meat quality, and gut microbiota in yellow-feathered chickens. The objective of this study was to investigate the regulatory mechanisms of TAU on chicken flavor by employing ultra-high-performance liquid chromatography tandem mass spectrometry (UPLC-MS/MS) and gas chromatography-mass spectrometry (GC–MS) to profile lipid molecular species and VOC.

## 2 Materials and methods

### 2.1 Animal ethics statement

The protocol for the animal experiment was authorized by the Animal Welfare and Ethics Committee of the Guangdong Academy of Agricultural Sciences, Institute of Animal Science (No. GAASISA-2024036/2024; approval date: Mar 6, 2024).

### 2.2 Chemicals

Taurine was provided by Guangzhou Huayu Biotech Co., Ltd. (107–35-7, Guangzhou, Guangdong, China), and its purity was >99% determined by high performance liquid chromatography (HPLC) analysis.

### 2.3 Animals and diets

Nine hundred 1-d-old female fast-growing *Lingnan* yellow-feathered chickens with similar initial body weight (38.58 ± 0.12 g) were acquired from Guangdong Wiz Group Co., Ltd. (Guangzhou, Guangdong, China) and randomly distributed into 5 groups. Each group consisted of 6 replicates, with 30 birds per replicate. The study was conducted over a period of 56 d. The first group served as the control (CON) and was fed a basal diet; the other 4 groups were fed experimental diets supplemented with 0.25, 0.50, 1.00, and 2.00 g/kg TAU (TAU250, TAU500, TAU1000, and TAU2000, respectively). The TAU doses were selected based on prior poultry studies demonstrating beneficial effects on antioxidant status and meat quality at these supplementation levels (Han et al., 2020; Huang et al., 2014; Lu et al., 2020). All birds were reared in floor pens (3.0 m × 1.2 m, starter period; 3.5 m × 2.2 m, growing and finishing period) with wood shavings (5 cm deep) and had *ad libitum* access to antibiotic-free feed and water. The room temperature was initially set at 32to35 °C for the first week and then reduced by 2 to3 °C per week to a final temperature of 26 °C. Room humidity was maintained between 50% and 60%.

The nutrient requirements for yellow-feathered chickens recommended by the Ministry of Agriculture of People’s Republic of China (2020) differed with the age of the birds (1–21 d, 22–42 d, and 43–56 d). The experimental ration formula and the specific nutritional concentrations are delineated in Table 1. Samples of feed collected during these periods were analyzed according to the methods of AOAC (2007). Briefly, the dry matter, crude protein, calcium, and total phosphorus were analyzed according to the methods 930.15, 990.03, 935.13, and 965.17, respectively.

**Table 1:** Ingredients and nutrient levels of the basal diet (%, dry matter basis).

Items	Age, d		
	1–21	22–42	43–56
**Ingredients**			
Corn	52.07	53.60	53.04
Wheat	5.00	10.00	15.00
Soybean meal	30.50	20.00	12.50
Rapeseed meal	2.50	4.00	6.00
Corn gluten meal	3.00	4.50	5.00
Soybean oil	2.28	3.40	4.40
L-Lysine hydrochloride (98.5%)	0.25	0.32	0.20
DL-Methionine (99%)	0.17	0.15	0.08
Limestone	1.25	1.18	1.15
Dicalcium phosphate	1.68	1.55	1.33
Sodium chloride	0.30	0.30	0.30
Premix^1^	1.00	1.00	1.00
Total	100.00	100.00	100.00
**Nutrients**^2^			
Organic matter	85.63	85.60	85.56
Apparent metabolizable energy, MJ/kg	12.38	12.80	13.20
Dry matter	88.52	88.28	87.84
Crude protein	21.48	19.56	18.03
Calcium	1.01	0.91	0.84
Total phosphorus	0.69	0.68	0.63
Non-phytate phosphorus	0.44	0.41	0.36
Lysine	1.29	1.15	0.96
Methionine	0.52	0.48	0.40
Methionine + cysteine	0.88	0.86	0.71
Threonine	0.86	0.81	0.67
Tryptophan	0.21	0.20	0.19
Isoleucine	0.77	0.69	0.68

1 The premix provided per kg of diet during 1 to 21 d: vitamin A, 12,000 IU; vitamin D_3_, 600 IU; vitamin E, 45 mg; vitamin K_3_, 6.0 mg; vitamin B_1_, 3.0 mg; vitamin B_2_, 9.0 mg; vitamin B_6_, 6.0 mg; vitamin B_12_, 0.03 mg; choline, 1300 mg; nicotinic acid, 60 mg; pantothenic acid, 18 mg; folic acid, 1.5 mg; biotin, 0.36 mg; Fe, 80 mg; Cu, 7.0 mg; Mn, 100 mg; Zn, 75 mg; I, 0.35 mg; Se, 0.15 mg. The premix provided per kg of diet during 22 to 42 d: vitamin A, 12,000 IU; vitamin D_3_, 500 IU; vitamin E, 45 mg; vitamin K_3_, 5.5 mg; vitamin B_1_, 3.0 mg; vitamin B_2_, 8.0 mg; vitamin B_6_, 5.0 mg; vitamin B_12_, 0.03 mg; choline, 1000 mg; nicotinic acid, 55 mg; pantothenic acid, 15 mg; folic acid, 1.25 mg; biotin, 0.36 mg; Fe, 70 mg; Cu, 7.0 mg; Mn, 80 mg; Zn, 70 mg; I, 0.35 mg; Se, 0.15 mg. The premix provided per kg of diet during 43 to 56 d: vitamin A, 12,000 IU; vitamin D_3_, 500 IU; vitamin E, 45 mg; vitamin K_3_, 5.0 mg; vitamin B_1_, 2.5 mg; vitamin B_2_, 7.5 mg; vitamin B_6_, 5.0 mg; vitamin B_12_, 0.03 mg; choline, 1000 mg; nicotinic acid, 50 mg; pantothenic acid, 15 mg; folic acid, 1.25 mg; biotin, 0.36 mg; Fe, 64 mg; Cu, 7.0 mg; Mn, 78 mg; Zn, 68 mg; I, 0.35 mg; Se, 0.15 mg.

2 Dry matter, crude protein, total calcium, and total phosphorus were measured value. Total lysine, methionine, methionine + cysteine, threonine, isoleucine, non-phytate phosphorus, organic matter, and metabolizable energy were calculated values according to China Feed Database (2020).

### 2.4 Growth performance

Body weight and feed intake were measured on a per-pen basis at 1, 21, 42, and 56 d post–hatch. These data were utilized to calculate the average daily gain (ADG), average daily feed intake (ADFI), and feed to gain ratio (F/G). Mortality was recorded daily to calculate the mortality rate.

### 2.5 Carcass traits and tissue samples preparation

On d 56, 2 birds per replicate with ADG close to the replicate mean were chosen and then stunned and euthanized through exsanguination. The carcass, semi-eviscerated carcass, eviscerated carcass, pectoral and thigh muscle weights were recorded for subsequent analysis and calculations of the percentages of them. The amount of abdominal adipose tissue was measured to estimate the proportion of abdominal fat. The left side breast muscle of the carcass was cut off as samples to measure the meat quality. To determine the composition of muscle fatty acids, a sample of 200 g from the left breast muscle tissue was frozen at −20 °C. In addition, breast muscle samples from the right side of the carcass were frozen in liquid nitrogen and used for RNA extraction and lipid metabolite measurement. Cecal contents were gathered in sterile microcentrifuge tubes and stored at −80 °C for the subsequent gut microbiota analysis.

### 2.6 Meat quality

For meat quality analysis, 12 birds were randomly selected from each group. Postmortem pH was measured at 15 min and 24 h after slaughter using a calibrated pH meter (Testo 205, Testo SE & Co. KGaA, Lenzkirch, Germany) by direct insertion into breast muscle at three different locations. Meat color, including lightness (*L*∗), redness (*a*∗), and yellowness (*b*∗), was measured in triplicate at 45 min and 24 h postmortem using a handheld CR-300 chromameter (Minolta Camera Co., Ltd., Osaka, Japan). To determine drip loss, breast muscle samples were sealed in pre-weighed plastic bags and stored for 24 h at 4 °C. After removal, excess surface moisture was absorbed using filter paper, and samples were reweighed. Drip loss was expressed as the percentage of weight loss. Meat tenderness was evaluated via Warner-Bratzler shear force measurement using an LRX texture analyzer (Model LRX, Lloyd Instruments Ltd., Fareham, UK). Briefly, breast muscle samples weighing approximately 300 g were heated in water at 80 °C until the central inner temperature reached 75 °C. Once the samples reached room temperature, they were precisely sliced into separate sections, each measuring 1 × 1 × 1 cm, with the cuts aligned parallel to the muscle fiber orientation for subsequent analysis.

### 2.7 Intramuscular fat content

The intramuscular fat (IMF) content in pectoral muscles was determined by the Soxhlet extraction technique using automatic fat analyzer (XT151, Ankom Technology Corporation, Macedon, NY, USA), as described by Folch et al. (1957). The IMF content was expressed as a percentage of muscle weight.

### 2.8 Fatty acid composition

For the separation of fatty acid methyl esters, a gas chromatography system using an Agilent DB-1 column (30 m × 0.25 mm, 0.25 μm; Agilent Technologies Inc., Folsom, CA, USA) was utilized. The identification of fatty acids was conducted with a QP2010-Plus GC–MS system (Shimadzu Corporation, Kyoto, Japan) based on their retention times. Additionally, the quantification of total fatty acids was carried out to verify the percentage composition of the identified fatty acids.

### 2.9 Activities of metabolic enzymes

The enzymatic activities of malate dehydrogenase (MDH; A021-2-1), succinate dehydrogenase (SDH; A022-1-1), isocitrate dehydrogenase (IDH; H311-1-2), lactate dehydrogenase (LDH; A020-2-2), and contents of protein carbonyl (PCO; A087-1-2), and malondialdehyde (MDA; A003-1-2) in breast muscle tissue were assayed using commercial assay kits from Nanjing Jiancheng Bioengineering Institute (Nanjing, Jiangsu, China) and measured with a microplate reader (SpectraMax, Molecular Devices (Shanghai) Corporation, Shanghai, China).

### 2.10 Untargeted lipidomic analysis

A total of 36 breast muscle tissue samples, with 12 from each group (CON, TAU500, and TAU2000), were selected for lipidomic analysis. The analysis was performed utilizing ultra-high-performance liquid chromatography coupled with a quadrupole-Orbitrap tandem mass spectrometer (UPLC-Q-Orbitrap MS/MS) manufactured by Thermo Fisher Scientific Inc. (Waltham, MA, USA). In this analysis, a 50 mg sample was homogenized in 500 μL of chloroform/methanol (2:1, v/v). The sample was combined with 150 μL of aqueous solution and vortexed for 2 min. Subsequently, it was incubated at 4 °C for 10 min. The homogenized sample was centrifuged at 4 °C and 3000 × *g* for 15 min. The chloroform layer was collected after phase separation, with equal aliquots pipetted into pre-chilled 1.5 mL tubes. Samples were then dried under a gentle nitrogen stream and preserved at −80 °C for long-term storage. The pellet was dissolved in 200 μL chloroform/methanol (2:1, v/v) containing internal standards, (phosphatidylcholine [14:0/14:0], phosphatidylglycerol [14:0/14:0], and fatty acid [18:0], each at 2 μg/mL) to facilitate further analysis. Aliquots from the treated samples were pooled to prepare quality control samples. Analysis was conducted using an Ultimate 3000 UPLC system coupled with a Q-Exactive Orbitrap mass spectrometer (Thermo Fisher Scientific Inc., Waltham, MA, USA), operated in both positive and negative ionization modes employing heated electrospray ionization (HESI).

The separation of lipids was conducted using a Waters Cortecs C18 column (2.1 × 100 mm, 1.6 μm; Waters Corporation, Milford, MA, USA) with a binary mobile phase: (A) 60% acetonitrile/water and (B) isopropyl alcohol/10% acetonitrile, both with 10 mM ammonium acetate. The gradient elution (0.3 mL/min flow rate) progressed from 30% B to 98% B over 14 min, followed by a 2-min wash at 100% B and re-equilibration (5 μL injection volume). Data were collected using mass spectrometry (MS/MS; Thermo Fisher Scientific Inc., Waltham, MA, USA) with a spray voltage of 3200 V in positive mode and 2800 V in negative mode. The auxiliary flow of 10 arbitrary was applied, with the capillary temperature consistently held at 320 °C during operation, with a mass range of 240 to 2000 *m*/*z* for positive mode and 200 to 2000 *m*/*z* for negative mode. The full scan resolution was set at 70,000, and the fragmentation spectra were acquired at a resolution of 17,500 with a duty cycle of 1.2 s. Lipid identification, including matching precursor ions to their characteristic fragment ions, was carried out using Lipid Search software version 4.2 (Mitsui Knowledge Industry Co., Ltd., Tokyo, Japan). Relative lipid concentrations were determined by normalizing chromatographic peak areas to corresponding internal standards.

### 2.11 Characterization of volatile compounds

Breast muscle volatiles were profiled using headspace solid-phase microextraction (HS-SPME) coupled with GC–MS (Shimadzu, Kyoto, Japan), featuring a InertCap WAX capillary column (60 m × 0.25 mm × 0.25 μm; GL Sciences Inc., Tokyo, Japan), as outlined by a previous study (Wen et al., 2019). Samples from 12 birds per group (2 birds from each replicate) were analyzed. For quantification, 5 g of homogenized breast tissue was spiked with 0.5 μg internal standard (2-methyl-3-heptanone, 0.05 μg/μL in methanol) prior to extraction. This mixture was transferred to a sealed headspace vial constructed from polytetrafluoroethylene (PTFE) and silicone. After a 20 min incubation at 55 °C, extraction was performed using a 75 μm divinylbenzene/carboxen/polydimethylsiloxane (DVB/CAR/PDMS) fiber, while maintaining a temperature of 55 °C for an additional 40 min. The extraction head underwent thermal desorption at a temperature of 250 °C for 3 min. To analyze the VOC, the GC–MS system utilized the aforementioned InertCap WaX capillary column (GL Sciences Inc., Tokyo, Japan). Analyses were performed using helium carrier gas (1.0 mL/min flow rate) with the ion source at 280 °C and a mass scan range of 30 to 450 *m*/*z*. Qualitative analysis of VOC was undertaken using the National Institute of Standards and Technology (NIST; Gaithersburg, MD, USA)14 mass spectral library for comparison of deconvoluted mass spectra. The linear retention index (LRI) was determined by analyzing a series of n-alkanes (C6–C20) under the same chromatographic conditions. Semi-quantification of VOC was performed by calculating their concentrations relative to the recovery rate of the internal standard.

### 2.12 Gut microbiota sequencing

A total of 36 cecal content samples of 12 per group (CON, TAU500, and TAU2000) were selected for gut microbiota sequencing. Cecal bacterial genomic DNA was isolated with the Mag-Bind Soil DNA Kit (M5635–2; Omega Bio-Tek, Inc., Norcross, GA, USA). DNA purity and concentration were assessed spectrophotometrically with the NanoDrop ND-2000 system (Thermo Fisher Scientific Inc., Waltham, MA, USA). Full-length 16S rRNA gene amplification (encompassing the V1–V9 hypervariable regions) was performed with the universal primers 27F and 1492R. The PCR products were purified with the QIAquick Gel Extraction Kit (28704; Qiagen GmbH, Hilden, Germany) and sequenced on the PacBio RS III platform (Majorbio Bio-Pharm Technology Co., Ltd., Shanghai, China). Bioinformatics analysis was performed following previously established methodologies (Campos et al., 2022).

### 2.13 Quantitative real-time PCR (qRT-PCR)

Total RNA from breast muscle tissue was isolated from 36 samples of 12 per group (CON, TAU500, and TAU2000) through TRIzol-based extraction (Invitrogen, Carlsbad, USA), and the resulting RNA was reverse-transcribed into cDNA using the PrimeScript RT system (RR037A; TaKaRa Bio Inc., Kusatsu, Shiga, Japan), incorporating a genomic DNA elimination step, according to the manufacturer's protocol. Quantitative real-time PCR was performed on a CFX96 Real-time PCR instrument (Bio-Rad Laboratories, Inc., Hercules, CA, USA). The synthesized primers were obtained from Sangong Bioengineering Co., Ltd. (Shanghai, China) and are listed in Supplementary Table S1. The full names of the abbreviations of all genes can be found in supplementary file abbreviations and full names of genes and proteins. The relative expression of the target genes in the breast sample was quantified by the 2^−ΔΔCt^ method (Livak and Schmittgen, 2001). β-Actin served as an internal control to ensure accurate normalization of the target gene expression.

### 2.14 Statistical analysis

The experimental data in Excel were preliminarily processed, and one-way analysis of variance (ANOVA) was carried out using the generalized linear model (GLM) procedure in SAS Version 9.4 (SAS Institute Inc., Cary, NC, USA) with the statistical model:

Yij=μ+Ji+eij,

where *Yij* is the dependent variable; *μ* is the overall mean; *Ji* is the fixed effect of treatment; and *eij* is the random residual error. Duncan's multiple range test was employed for multiple comparisons, with *P* < 0.05 indicating significant differences. Orthogonal polynomial contrasts were employed to evaluate the linear and quadratic effects to varying levels of TAU supplementation. The results are expressed as mean values accompanied by standard errors of the mean (SEM).

SIMCA version 15.0 (Umetrics AB, Umea, Sweden) was employed to conduct comprehensive chemometric analyses, including partial least squares discriminant analysis (PLS-DA) and orthogonal partial least squares discriminant analysis (OPLS-DA). The variable importance in projection (VIP) selection method of VIP >1 and |*P*(coor)| > 0.5 were selected to determine the most significant markers differentiating the groups. Pearson's correlation coefficient was used to evaluate the co-occurrence relationships among lipid metabolites, VOC, and intestinal microbial communities. A *P*-value less than 0.05 was considered indicative of statistical significance.

## 3 Results

### 3.1 Growth performance and carcass characteristics

Dietary TAU supplementation significantly increased the ADG and final body weight of yellow-feathered chickens from d 1 to 42 and d 1 to 56, compared with the CON group (*P* < 0.05) (Table 2). At the same time, the F/G was significantly decreased from d 1 to 42 and d 1 to 56 (*P* < 0.05). As shown in Supplementary Table S2, abdominal fat rate was lower in the TAU2000 group than in the CON group (*P* = 0.03). There were no significant differences in slaughter rate, eviscerated rate, breast muscle rate and thigh muscle rate between groups (*P* > 0.05).

**Table 2:** Effects of dietary taurine supplementation on growth performance of yellow-feathered chickens.

Items	Groups					SEM	*P*-value		
	CON	TAU250	TAU500	TAU1000	TAU2000		ANOVA	Linear	Quadratic
**1**–**21 d**									
Initial body weight, g	38.56	38.61	38.58	38.58	38.57	0.035	0.910	0.610	0.684
Weight at 21 d, g	495.53	487.12	484.92	485.91	488.64	6.029	0.745	0.166	0.527
ADFI, g	34.36	33.89	33.65	34.13	33.71	0.287	0.744	0.165	0.524
ADG, g/d	21.76	21.36	21.25	21.3	21.43	0.346	0.575	0.116	0.633
F/G	1.58	1.59	1.58	1.60	1.57	0.013	0.566	0.826	0.694
Mortality rate, %	0.00	0.00	0.00	0.00	0.00	–	–	–	–
**1**–**42 d**									
Weight at 42 d, g	1332.4^b^	1361.1^ab^	1399.6^a^	1399.2^a^	1404.6^a^	18.37	0.040	0.073	0.096
ADFI, g	62.11	62.13	62.01	62.82	62.58	0.579	0.836	0.839	0.160
ADG, g/d	30.81^b^	31.49^ab^	32.41^a^	32.39^a^	32.52^a^	0.437	0.040	0.073	0.096
F/G	2.02^a^	1.97^ab^	1.92^b^	1.94^b^	1.92^b^	0.018	0.004	0.003	0.079
Mortality rate, %	0.00	0.00	0.00	0.00	0.76	0.005	0.567	0.262	1.000
**1**–**56 d**									
Weight at 56 d, g	1892.7^b^	1952.4^a^	1977.7^a^	2004.7^a^	1986.3^a^	18.67	0.003	0.020	0.003
ADFI, g	79.07	78.41	77.33	78.57	77.78	0.976	0.741	0.196	0.934
ADG, g/d	33.11^b^	34.18^a^	34.63^a^	35.11^a^	34.78^a^	0.333	0.003	0.020	0.003
F/G	2.39^a^	2.29^b^	2.23^b^	2.34^b^	2.24^b^	0.023	<0.001	0.002	0.010
Mortality rate, %	0.00	0.00	0.00	0.00	0.76	0.005	0.567	0.262	1.000

ADFI = average daily feed intake; ADG = average daily gain; F/G = feed to gain ratio; SEM = standard error of the mean; CON = basal diet group; TAU250 = basal diet supplementation with 0.25 g/kg taurine group; TAU500 = basal diet supplementation with 0.50 g/kg taurine group; TAU1000 = basal diet supplementation with 1.00 g/kg taurine group; TAU2000 = basal diet supplementation with 2.00 g/kg taurine group.

Means within a row with different superscript letters are significantly different (*P* < 0.05). Each value represents the mean of 6 replicates (30 chickens per replicate) per treatment.

### 3.2 Meat quality

As illustrated in Table 3, dietary supplementation with 0.25 g/kg TAU led to a significant reduction in drip loss_24 h_ (*P* < 0.05). Dietary supplementation with 1.0 g/kg TAU also decreased drip loss_24 h_ and increased pH_24 h_ (*P* < 0.05). Dietary supplementation with 2.0 g/kg TAU increased pH_24 h_, *a*∗ value and decreased drip loss_24 h_ (*P* < 0.05). Furthermore, the drip loss_24 h_ in the TAU2000 group was significantly lower than that in the TAU250 group (*P* < 0.001). There were no differences in the meat quality traits, including *L*∗, *b*∗, pH_45 min_, and shear force, among the CON group and TAU supplemented groups (*P* > 0.05). The contents of the IMF showed a tendency to increase with dietary TAU inclusion, compared with the CON group (*P* = 0.069).

**Table 3:** Effects of dietary taurine supplementation on meat quality of yellow-feathered chickens.

Items	Groups					SEM	*P*-value		
	CON	TAU250	TAU500	TAU1000	TAU2000		ANOVA	Linear	Quadratic
L∗_45 min_	46.86	45.78	46.63	45.58	46.32	0.334	0.108	0.056	0.007
a∗_45 min_	13.44^b^	13.62^ab^	13.45^b^	13.88^ab^	14.79^a^	0.151	0.021	0.113	0.002
b∗_45 min_	7.04	8.79	7.78	7.93	8.17	0.219	0.179	0.131	0.063
pH _45 min_	6.02	6.04	6.08	6.06	6.02	0.019	0.851	0.531	0.692
pH _24 h_	5.83^b^	5.81^b^	5.80^b^	5.92^a^	5.94^a^	0.011	<0.001	0.007	<0.001
L∗_24 h_	49.61	48.73	51.13	48.34	49.52	0.348	0.104	0.165	0.069
a∗_24 h_	10.78	10.64	10.00	11.10	10.79	0.170	0.335	0.111	0.239
b∗_24 h_	7.54	9.04	8.55	8.34	8.97	0.202	0.129	0.060	0.066
Drip loss_24 h_, %	3.97^a^	3.30^b^	2.72^c^	2.40^cd^	2.06^d^	0.002	<0.001	<0.001	<0.001
Shear force, N	20.70	23.90	21.06	16.56	18.61	1.436	0.580	0.431	0.628
IMF, %	1.03	1.08	1.12	1.26	1.31	0.056	0.069	0.018	0.503

L∗ = lightness; a∗ = redness; b∗ = yellowness; IMF = intramuscular fat; SEM = standard error of the mean; CON = basal diet group; TAU250 = basal diet supplementation with 0.25 g/kg taurine group; TAU500 = basal diet supplementation with 0.50 g/kg taurine group; TAU1000 = basal diet supplementation with 1.00 g/kg taurine group; TAU2000 = basal diet supplementation with 2.00 g/kg taurine group.

Means within a row with different superscript letters are significantly different (*P* < 0.05). Each value represents the mean of 6 replicates (30 chickens per replicate) per treatment.

### 3.3 Fatty acid composition

The fatty acid composition of breast muscle is shown in Table 4. Compared with the CON group, the proportion of C18:0 was decreased by 1.00 and 2.00 g/kg TAU supplementation (*P* = 0.037). Dietary supplementation with 1.00 and 2.00 g/kg TAU resulted in higher monounsaturated fatty acids (MUFA) and C18:2n-6, compared with the CON group (*P* < 0.05). There were no significant differences in polyunsaturated fatty acids (PUFA) and n-6/n-3 ratio between groups (*P* > 0.05).

**Table 4:** Effects of dietary taurine supplementation on fatty acid composition of breast muscle in yellow-feathered chickens (%, total fatty acid).

Items	Groups					SEM	*P*-value		
	CON	TAU250	TAU500	TAU1000	TAU2000		ANOVA	Linear	Quadratic
C14:0	0.476	0.554	0.477	0.448	0.492	0.0311	0.199	0.304	0.492
C15:0	1.898	1.181	2.062	2.013	1.323	0.4729	0.562	0.740	0.251
C16:0	22.59	22.28	22.27	22.26	22.19	0.505	0.983	0.766	0.803
C17:0	0.116	0.119	0.122	0.124	0.124	0.0068	0.905	0.582	0.641
C18:0	7.484^a^	7.227^ab^	7.467^a^	6.877^b^	6.612^b^	0.1693	0.037	<0.001	0.309
SFA^1^	32.56	31.60	32.16	31.72	30.74	1.008	0.802	0.785	0.253
C14:1	0.121	0.116	0.119	0.122	0.128	0.0089	0.544	0.458	0.125
C16:1	3.217	3.861	3.063	2.983	3.592	0.3545	0.378	0.599	0.257
C18:1	26.45^b^	27.43^ab^	28.22^ab^	29.91^a^	29.08^a^	1.326	0.041	0.025	0.505
C20:1	0.318	0.367	0.308	0.307	0.317	0.0236	0.251	0.601	0.148
MUFA^2^	29.91^b^	31.78^ab^	30.99^ab^	32.49^a^	33.94^a^	1.636	0.037	<0.001	0.429
C18:2n-6	24.92^b^	25.79^ab^	25.00^ab^	26.63^a^	26.72^a^	0.626	0.039	0.008	0.424
C18:3n-6	0.164	0.201	0.159	0.151	0.189	0.0156	0.166	0.404	0.217
C18:3n-3	1.658	1.527	1.575	1.808	1.845	0.1377	0.409	0.745	0.335
C20:2	0.453	0.413	0.454	0.511	0.417	0.0655	0.839	0.615	0.782
C20:3	0.563	0.478	0.664	0.715	0.524	0.0979	0.419	0.921	0.539
C20:4	3.148	2.303	4.116	4.296	2.771	0.7229	0.267	0.798	0.294
C20:5	0.184	0.239	0.247	0.258	0.274	0.0729	0.956	0.707	0.877
C22:6	0.330	0.239	0.404	0.496	0.549	0.0872	0.145	0.647	0.282
PUFA^3^	32.35	32.22	31.91	31.87	31.72	0.851	0.983	0.669	0.983
n-6 PUFA	30.18	29.94	30.09	29.94	29.62	0.804	0.990	0.865	0.928
n-3 PUFA	2.172	2.005	2.226	2.562	2.668	0.0658	0.276	0.230	0.569
n-6/n-3 ratio	13.09	13.74	13.19	12.91	12.79	0.324	0.299	0.303	0.771

SFA = saturated fatty acids; MUFA = monounsaturated fatty acids; PUFA = polyunsaturated fatty acids; n-6/n-3 ratio = ratio of n-6 PUFA to n-3 PUFA; SEM = standard error of the mean; CON = basal diet group; TAU250 = basal diet supplementation with 0.25 g/kg taurine group; TAU500 = basal diet supplementation with 0.50 g/kg taurine group; TAU1000 = basal diet supplementation with 1.00 g/kg taurine group; TAU2000 = basal diet supplementation with 2.00 g/kg taurine group.

Means within a row with different superscript letters are significantly different (*P* < 0.05). Each value represents the mean of 6 replicates (2 chickens per replicate) per treatment.

1 The SFA including C10:0, C12:0, C13:0, C14:0, C15:0, C16:0, C17:0, and C18:0 are analyzed values.

2 The MUFA including C14:1, C15:1, C16:1n-7, C17:1n-7, C18:1n-9, and C20:1n-9 are analyzed values.

3 The PUFA including C18:2n-6, C20:2n-6, C18:3n-6, C18:3n-3, C20:3n-3, C20:3n-6, C20:5n-3, C22:2n-3, and C22:6n-3 are analyzed values.

### 3.4 Oxidation index analysis

As shown in Table 5, no significant differences were found in MDH and PC levels between groups (*P* > 0.05). However, dietary supplementation with TAU resulted in significantly higher SDH and IDH activities and a lower LDH activity compared with the CON group (*P* < 0.05). Dietary 2.0 g/kg TAU supplementation significantly reduced MDA content in breast muscle compared with the CON group (*P* = 0.015).

**Table 5:** Effects of dietary taurine supplementation on energy metabolism-related enzymes activities (U/mg prot) of breast muscle in yellow-feathered chickens.

Items	Groups					SEM	*P*-value		
	CON	TAU250	TAU500	TAU1000	TAU2000		ANOVA	Linear	Quadratic
LDH	112.08^a^	93.67^b^	91.77^b^	92.16^b^	74.99^c^	6.035	<0.001	0.003	<0.001
MDH	0.64	0.65	0.69	0.72	0.85	0.061	0.429	0.848	0.369
SDH	0.68^b^	0.89^ab^	0.95^ab^	1.28^a^	1.49^a^	0.218	0.046	0.012	0.254
IDH	2.64^b^	6.56^a^	7.23^a^	8.80^a^	7.94^a^	1.023	0.002	0.024	0.120
MDA	0.36^a^	0.38^a^	0.35^ab^	0.32^ab^	0.26^b^	0.029	0.015	0.022	0.056
PCO	655.7	589.7	806.9	764.1	619.9	35.03	0.677	0.503	0.146

LDH = lactic dehydrogenase; MDH = malic dehydrogenase; SDH = succinate dehydrogenase; IDH = isocitrate dehydrogenase; MDA = malondialdehyde; PCO = protein carbonyl; SEM = standard error of the mean; CON = basal diet group; TAU250 = basal diet supplementation with 0.25 g/kg taurine group; TAU500 = basal diet supplementation with 0.50 g/kg taurine group; TAU1000 = basal diet supplementation with 1.00 g/kg taurine group; TAU2000 = basal diet supplementation with 2.00 g/kg taurine group.

Means within a row with different superscript letters are significantly different (*P* < 0.05). Each value represents the mean of 6 replicates (12 chickens per replicate) per treatment.

### 3.5 Untargeted lipidomic profile

A total of 1493 lipids belonging to 17 subclasses were detected, with 1002 and 491 lipids identified in positive and negative electrospray ionization modes, respectively. As shown in (Fig. S1A), the lipid composition included 545 (36.53%) triglycerides (TG), 275 (18.43%) phosphatidylcholines (PC), 221 (14.81%) diglycerides (DG), 216 (14.48%) phosphatidylethanolamines (PE), 53 (3.55%) sphingomyelins (SM), 48 (3.22%) lysophosphatidylcholines (LPC), 31 (2.08%) ceramide (CER), 26 (1.74%) phosphatidylinositols (PI), and other minor lipid metabolites. The score plots from OPLS-DA analysis of lipid metabolites in both positive and negative ion modes are depicted in Fig. 1A and B, respectively. The three groups exhibit a separation trend in both ion modes. In this study, 376 significant differential metabolites were detected by screening, including 254 metabolites in the positive ion mode and 121 metabolites in the negative ion mode, which contained 81 TG, 79 PE, 72 PC, 65 DG, 19 CER, 18 SM, 10 LPC, 6 PI, 5 phosphatidylglycerol (PG), 4 diglycosylceramide (CerG2), 4 monoglyceride (MG), 4 phosphatidylserine (PS), 2 triglycosylceramide (CerG3), 2 ganglioside m3 (GM3), 2 lysophosphatidylglycerol (LPG), 2 lysophosphatidylserine (LPS), and 1 lysophosphatidylethanolamine (LPE) (Fig. S1B). A heatmap depicting the top 20 VIP differential lipid metabolites was generated based on relative abundance, as shown in Fig. 1C. These metabolites include 6 types of PE, 5 types of PC, 2 types of PI, 4 types of TG, and one type of LPC, CER, and CerG2. Notably, dietary 2.0 g/kg TAU supplementation significantly increased 4 PE in PE (40:3p) + Na, PE (18:0p/22:3) + H, PE (16:0p/18:1) + H, and PE (20:2/20:2) + H; 2 PC in PC (40:8p) + H, and PC (16:0/8:0) + H; 3 TG in TG (16:2/18:3/18:3) + Na, TG (18:4/14:1/18:2) + NH_4_, and TG (55:4p) + K; 2 PI in PI (20:2/20:2) + K and PI (18:0/20:3) + K; 1 LPC (18:0P) + H (*P* < 0.05).

**Fig. 1:**
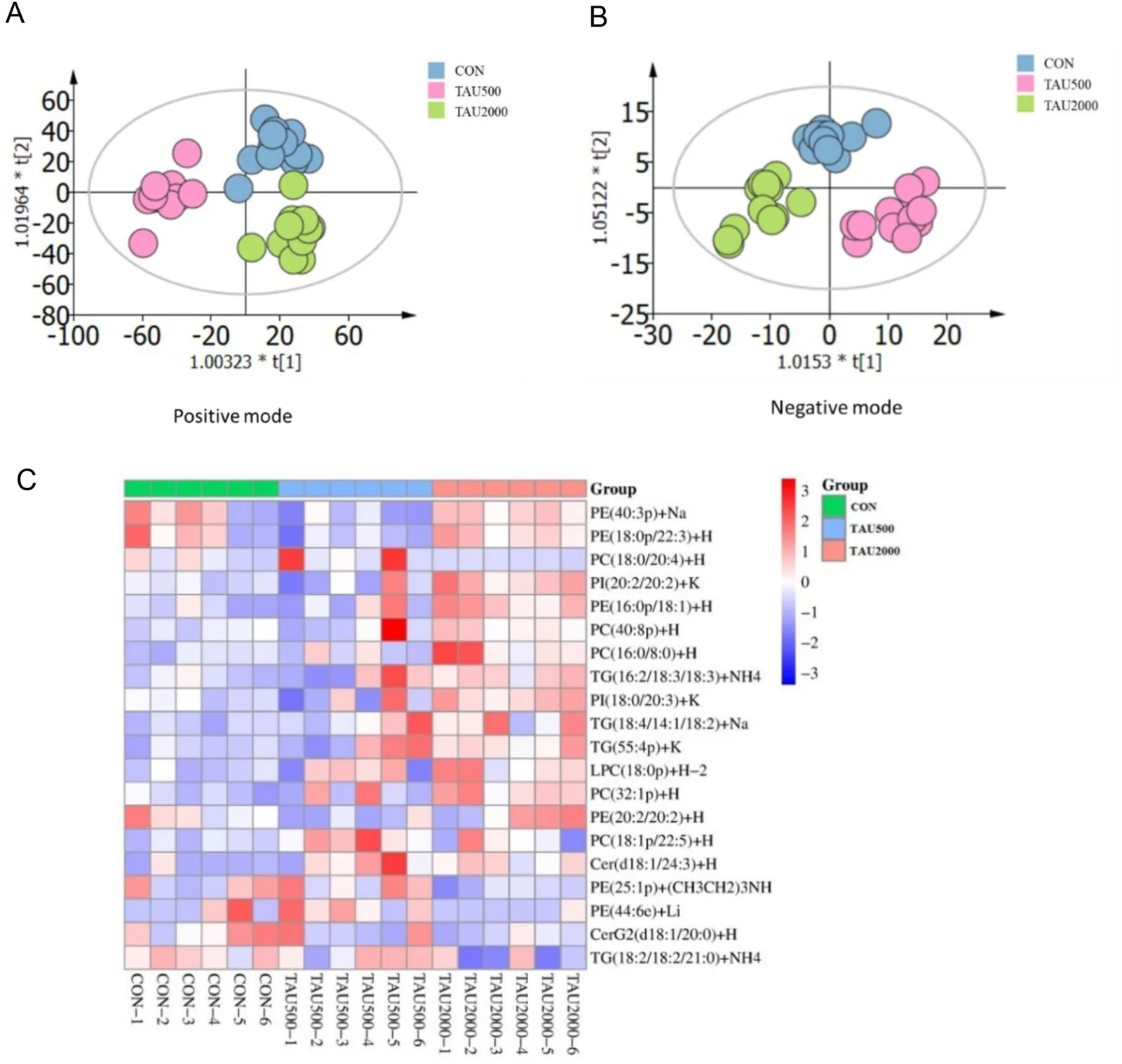
Effects of different dietary taurine supplementation on lipid metabolites of breast muscle in broiler chickens (*n* = 12). (A) Orthogonal partial least squares discriminant analysis (OPLS-DA) score of lipid metabolites in positive ion mode. The scores t[1] and t[2] represent the sample scores on the first predictive component and the second orthogonal component, respectively; t[1] captures between-group variation, whereas t[2] reflects within-group variation. (B) The OPLS-DA score of lipid metabolites in negative ion mode. (C) The abundance of top 20 differential lipid metabolites by variable importance in projection (VIP) ranking. CON = basal diet group; TAU500 = basal diet supplementation with 0.50 g/kg taurine group; TAU2000 = basal diet supplementation with 2.00 g/kg taurine group. PE = phosphatidylethanolamines; PC = phosphatidylcholines; PI = phosphatidylinositols; TG = triglycerides; LPC = lysophosphatidylcholines; Cer = ceramide; CerG2 = diglycosylceramide.

### 3.6 Volatile organic compounds composition

The PLS-DA and OPLS-DA analysis were further used to identify the characteristic distribution of VOC in the breast muscle of chickens fed with TAU. Although PLS-DA and OPLS-DA showed no significant differences in terms of fit or reliability, OPLS-DA presented a better classification of VOC in the breast muscle of chickens fed with TAU (Fig. 2). The main VOC components in breast muscle are displayed in Table 6, The contents of 3-methylbutanal, (E)-2-octanal, ethyl acetate, ethyl elaidate, ethyl lactate, ethyl myristate, ethyl palmitate, ethyl 9,12-octadecadienoic acid, methyl dodecanoate, and nonadecane in the TAU500 and TAU2000 groups were significantly higher than those in the CON group (*P* < 0.05). However, the contents of pentanal, hexanal, 2-nonenal, nonanal, 2-propanone, 3-methylbutyrolactone, pentadecane, 2-amylfuran, and pyrazine in TAU500 and TAU2000 were lower than those in the CON group (*P* < 0.05).

**Fig. 2:**
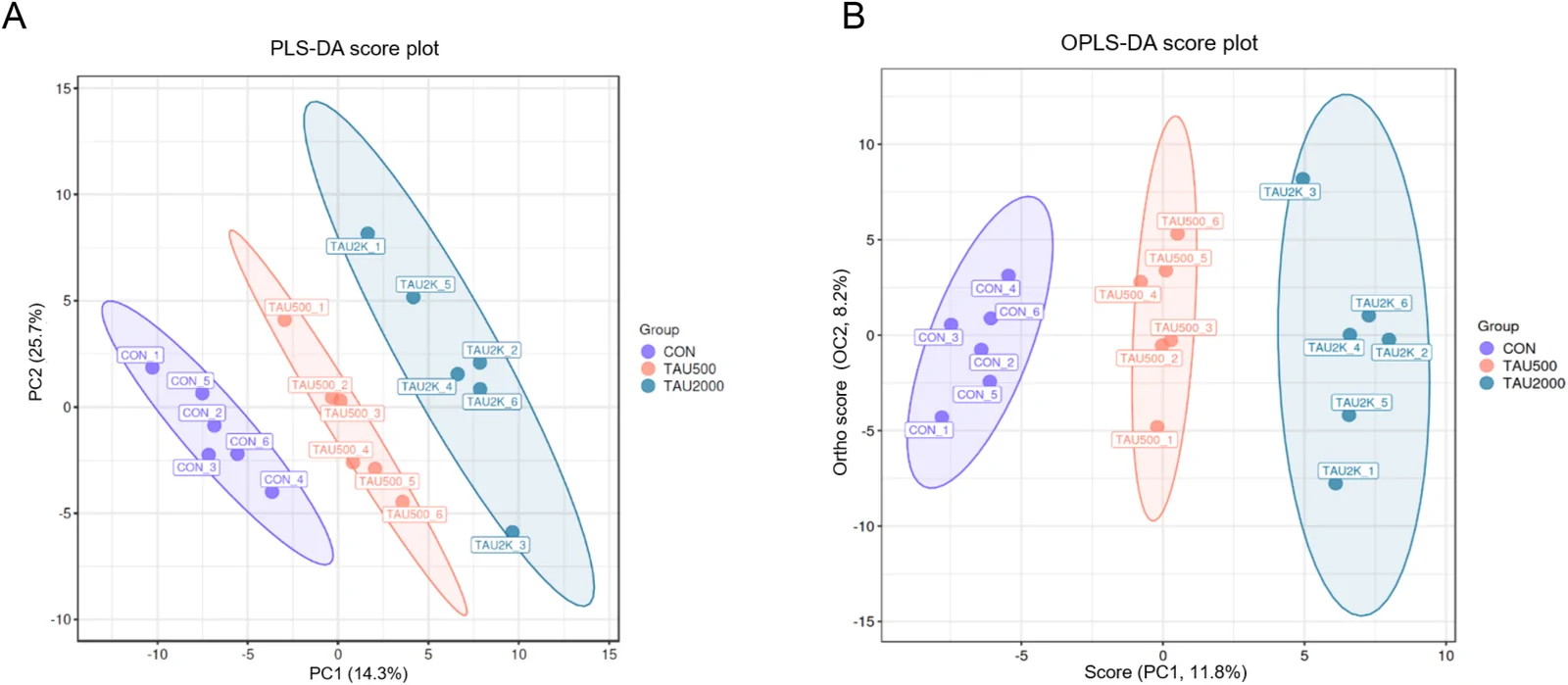
Effects of different dietary taurine supplementation on volatile organic compounds (VOC) of breast muscle in broiler chickens (*n* = 6). (A) Score plots of partial least-squares discriminant analysis (PLS-DA) was derived from gas chromatography-mass spectrometry (GC–MS) data showing the VOC differences of breast muscle samples among different treatment groups of broiler chickens. (B) Score plots of orthogonal partial least squares discriminant analysis (OPLS-DA) was derived from GC–MS data showing the VOC differences of breast muscle samples among different treatment groups of broiler chickens. CON = basal diet group; TAU500 = basal diet supplementation with 0.50 g/kg taurine group; TAU2000 = basal diet supplementation with 2.00 g/kg taurine group.

**Table 6:** Effects of dietary taurine supplementation on volatile organic compounds (VOC) composition of breast muscle in broiler chickens.

No.	Compounds	RT, min	Molecular formula	Peak intensity			SEM	*P-*value
				CON	TAU500	TAU2000		
1	2-Methyl-2-butenal	9.17	C_5_H_8_O	86.83^b^	98.26^b^	125.81^a^	17.558	0.023
2	3-Methylbutanal	5.13	C_5_H_10_O	136.66^b^	187.24^a^	189.36^a^	12.371	0.014
3	2-Methylbutyraldehyde	5.06	C_5_H_10_O	29.88^b^	52.09^ab^	63.57^a^	7.588	0.021
4	Pentanal	6.34	C_5_H_10_O	1344.2^a^	940.0^b^	680.7^c^	212.94	0.012
5	Hexanal	8.78	C_6_H_12_O	10357^a^	6691^b^	5746^c^	395.8	0.016
6	Heptanal	11.63	C_7_H_14_O	405.5^a^	249.7^ab^	205.4^b^	73.48	0.016
7	(E)-2-Octenal	18.38	C_8_H_14_O	133.3^b^	167.1^a^	189.7^a^	114.19	0.038
8	2-Nonenal	21.10	C_9_H_16_O	87.40^a^	48.11^b^	32.72^b^	21.494	0.021
9	Nonanal	17.38	C_9_H_18_O	473.6^a^	394.9^b^	385.1^b^	72.18	0.045
10	Decaldehyde	20.12	C_10_H_20_O	89.99^a^	71.83^a^	68.92^b^	12.192	0.042
11	Tetradecanal	29.89	C_14_H_28_O	70.74^a^	47.69^ab^	19.85^b^	12.192	0.032
12	Pentadecanal	32.05	C_15_H_30_O	103.24^a^	89.87^a^	58.07^b^	9.722	0.014
13	Ethanol	5.35	C_2_H_6_O	409841^b^	490822^ab^	511890^a^	378.5	0.006
14	Acetol	15.02	C_3_H_6_O_2_	38.39^a^	34.10^a^	23.69^b^	6.581	0.029
15	1-Octen-3-ol	18.68	C_8_H_16_O	1145.8^b^	1419.7^ab^	1694.8^a^	170.61	0.011
16	1-Octanol	21.43	C_8_H_18_O	129.08^a^	100.03^ab^	57.46^b^	26.879	0.019
17	2-Ethyl-1-hexanol	19.72	C_8_H_18_O	1527.9^a^	1436.5^a^	1084.0^b^	142.90	0.010
18	2-Propanone	6.28	C_3_H_6_O	253.48^a^	150.69^b^	74.03^c^	18.602	0.012
19	2-Heptanone	11.51	C_7_H_14_O	152.5^b^	217.8^ab^	262.8^a^	72.19	0.032
20	2-Octanone	14.39	C_8_H_16_O	939.1^b^	1249.6^ab^	1628.0^a^	168.49	0.036
21	3-Octanone	13.53	C_8_H_16_O	37.81^a^	32.12^ab^	19.20^b^	10.836	0.035
22	Ethyl acetate	4.64	C_4_H_8_O_2_	255.4^c^	790.4^b^	1307.0^a^	264.95	0.042
23	Ethyl lactate	16.08	C_5_H_10_O_3_	881.2^b^	2036.2^a^	2084.4^a^	199.41	<0.001
24	Ethyl myristate	32.42	C_16_H_32_O_3_	31.58^c^	83.78^b^	145.44^a^	11.084	<0.001
25	Ethyl palmitate	36.32	C_18_H_36_O_3_	415.0^c^	773.4^b^	1182.3^a^	113.27	<0.001
26	Ethyl elaidate	40.64	C_20_H_38_O_2_	118.52^c^	225.01^b^	339.48^a^	34.459	0.002
27	Ethyl 9,12-octadecadienoic acid	41.78	C_20_H_36_O_2_	192.14^c^	351.37^b^	527.80^a^	47.951	<0.001
28	Ethyl octadec-9-enoate	40.82	C_20_H_38_O_2_	7.07^b^	16.16^ab^	22.20^a^	4.004	0.048
29	Methyl dodecanoate	28.17	C_14_H_28_O_2_	31.23^b^	60.99^a^	69.92^a^	19.418	0.048
30	2-Methylpentane	4.33	C_6_H_14_	21.27^b^	26.98^b^	36.19^a^	3.138	0.038
31	3-Methylbutyrolactone	23.26	C_5_H_8_O_2_	15.09^a^	7.81^b^	0.21^c^	2.048	<0.001
32	Octane	3.43	C_8_H_18_	21.99^b^	30.06^ab^	37.94^a^	10.226	0.036
33	2,4-Dimethylheptane	3.51	C_9_H_2_O	129.08^b^	166.72^b^	215.99^a^	33.581	0.022
34	2,6-Dimethylnonane	7.68	C_11_H_14_	54.68^b^	175.15^a^	149.39^ab^	32.222	0.044
35	5-Methyldodecane	12.75	C_13_H_18_	3.05^b^	4.21^b^	12.25^a^	3.512	0.011
36	Pentadecane	19.78	C_15_H_32_	211.22^a^	142.58^b^	70.69^c^	16.836	0.011
37	3-Methylhexadecane	25.25	C_17_H_36_	10.75^b^	12.16^b^	47.95^a^	11.256	0.029
38	Nonadecane	26.98	C_18_H_38_	31.93^c^	64.96^b^	102.39^a^	21.238	0.022
39	2-Amylfuran	12.90	C_9_H_14_O	364.6^a^	248.5^b^	216.8^b^	90.26	0.049
40	Pyrazine	12.33	C_4_H_4_N_2_	92.22^a^	62.92^b^	52.09^b^	23.481	0.031

RT = retention time; CON = basal diet group; TAU500 = basal diet supplementation with 0.50 g/kg taurine group; TAU2000 = basal diet supplementation with 2.00 g/kg taurine group; SEM = standard error of the mean.

Means within a row with different superscript letters are significantly different (*P* < 0.05). Each value represents the mean of 6 replicates (12 chickens per replicate) per treatment.

### 3.7 Intestinal microbiota structure

As illustrated in Table S3, the cecal microbiota of the TAU2000 group exhibited significant differences in α-diversity indices, including Ace, Chao, and Good's coverage compared with the CON group (*P* < 0.05). The principal coordinates analysis (PCoA) showed distinct dissociation clustering in β-diversity within groups (*P* = 0.002) (Fig. 3A). As illustrated in Fig. 3B, the phylum-level analysis identified the presence of Firmicutes, Bacteroidota, Deferribacterota, Proteobacteria, Actinobacteriota, Desulfobacterota, Campylobacterota, Synergistota, Cyanobacteria, Verrucomicrobiota, and other minor phyla. At the phylum level, dietary 2.00 g/kg TAU supplementation increased the abundance of Bacteroidota but decreased that of Firmicutes and the Firmicutes/Bacteroidota ratio, compared to the CON group (*P* < 0.05) (Fig. 3C). The relative abundances of the 20 most prevalent genera among groups are summarized in Fig. 3D and E. Dietary 2.00 g/kg TAU supplementation increased the abundance of *Bacteroides*, *Faecalibacterium* and *Enterococcus* and decreased *Streptococcus* and *Lachnoclostridium*, compared to the CON group (*P* < 0.05).

**Fig. 3:**
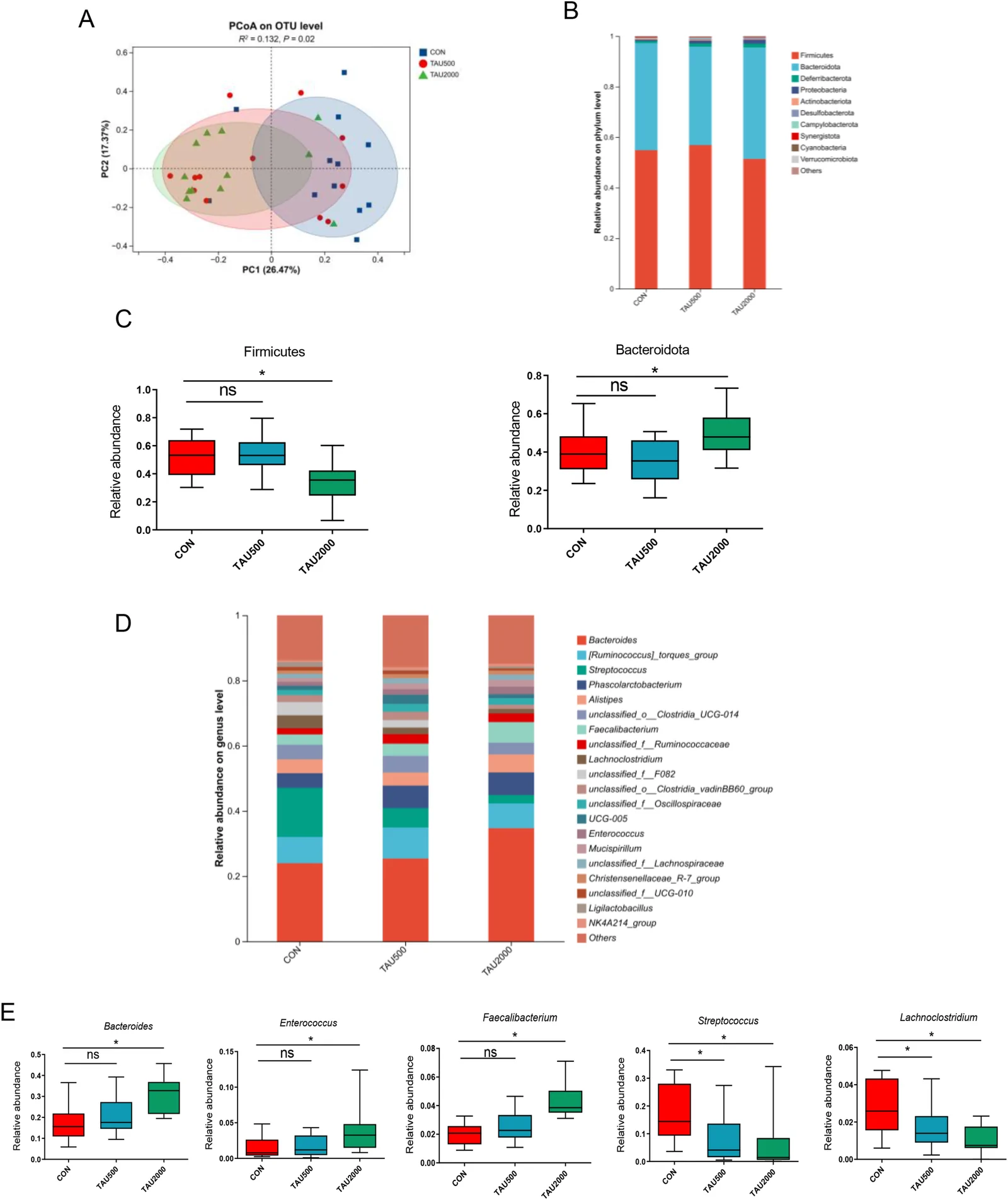
Effects of dietary taurine supplementation on the gut microbiota of broiler chickens (*n* = 12). (A) Principal coordinates analysis (PCoA) at the operational taxonomic unit (OTU) level. (B) Stacked bar chart showing the relative abundance of colonic bacteria, at the phylum level (top 10) in the different dietary groups. (C) The significant different colonic microbial phyla in the different dietary groups. (D) Stacked bar chart representing the relative abundance of colonic bacteria, at the genus level (top 20) in the different dietary groups. (E) The significant different colonic microbial genera in the different dietary groups. CON = basal diet group; TAU500 = basal diet supplementation with 0.50 g/kg taurine group; TAU2000 = basal diet supplementation with 2.00 g/kg taurine group. ∗ Indicates a statistically significant difference between groups at *P* < 0.05.

### 3.8 Expression of breast muscle lipid metabolism-related genes

As indicated in Fig. 4, dietary 2.00 g/kg TAU supplementation increased the mRNA expression levels of *ACACA*, *FABP3*, *SREBP1*, *PPARα*, while decreased the mRNA expression levels of *CPT1A*, *ACOX1*, and *ACSL5* in breast muscle compared to the CON (*P < 0.05*). No differences were found in the mRNA expression level of *FASN* between the groups (*P* > 0.05).

**Fig. 4:**
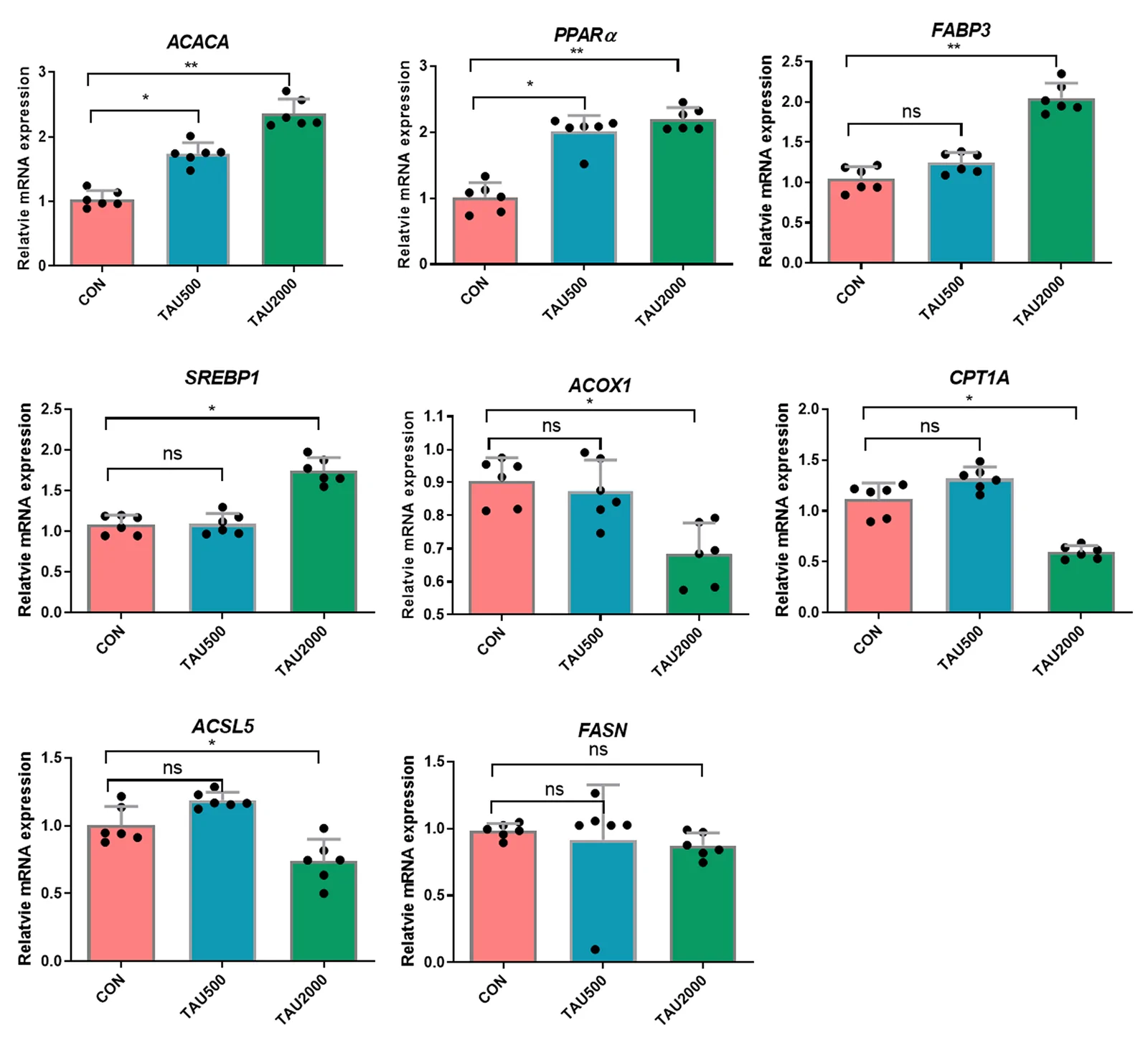
Effects of different dietary taurine supplementation on the relative gene expression of breast muscle in broiler chickens (*n* = 12). CON = basal diet group; TAU500 = basal diet supplementation with 0.50 g/kg taurine group; TAU2000 = basal diet supplementation with 2.00 g/kg taurine group. ∗ Indicates a statistically significant difference between groups at *P* < 0.05; ∗∗ indicates a higher statistically significant difference between groups at *P* < 0.01.

### 3.9 Correlation analysis between breast muscle lipid metabolites, volatile organic compounds, and intestinal microbial communities

The interactions of lipid metabolites, VOC, and the key cecal bacterial genera by Pearson's method demonstrated that the *Enterococcus* abundance exhibited a positive correlated with the content of PC (32:1p) + H, PC (16:0/8:0) + H, PE (16:0p/18:1) + H, and PI (20:2/120:2) + K (*P* < 0.05); *unclassified_o_Clostridia_UCG-014* was positively correlated with the content of CerG2 (d18:1/20:0) + H, PE (44:6e) + Li, PE (25:1p) + (CH3CH2)3NH, and PE (18:0/20:4) + H (*P* < 0.05); Christensenellaceae_R7_group was also positively correlated with the content of PE (18:0/20:4) + H in breast muscle (*P* < 0.05) (Fig. 5A). Furthermore, the *Faecalibacterium* abundance was correlated positively with 2-methyl-2-butenal, (E)-2-octenal, ethanol, 1-octen-3-ol, 2-octanone, ethyl acetate, ethyl elaidate, ethyl lactate, ethyl myristate, ethyl palmitate, ethyl 9,12-octadecadienoic acid, nonadecane, and octane (*P* < 0.05). *Enterococcus* abundance showed significant positive correlations with 2-methylbutyraldehyde, (E)-2-octenal, ethyl myristate, ethyl palmitate, ethyl elaidate, ethyl 9,12-octadecadienoic acid, 2-methylpentane, and octane (*P* < 0.05), whereas *Streptococcus* and *Lachnoclostridium* showed significantly negative correlations with ethyl acetate, ethyl elaidate, ethyl lactate, ethyl mmyristate, ethyl palmitate, ethyl 9,12-octadecadienoic acid, and octane (*P* < 0.05) (Fig. 5B).

**Fig. 5:**
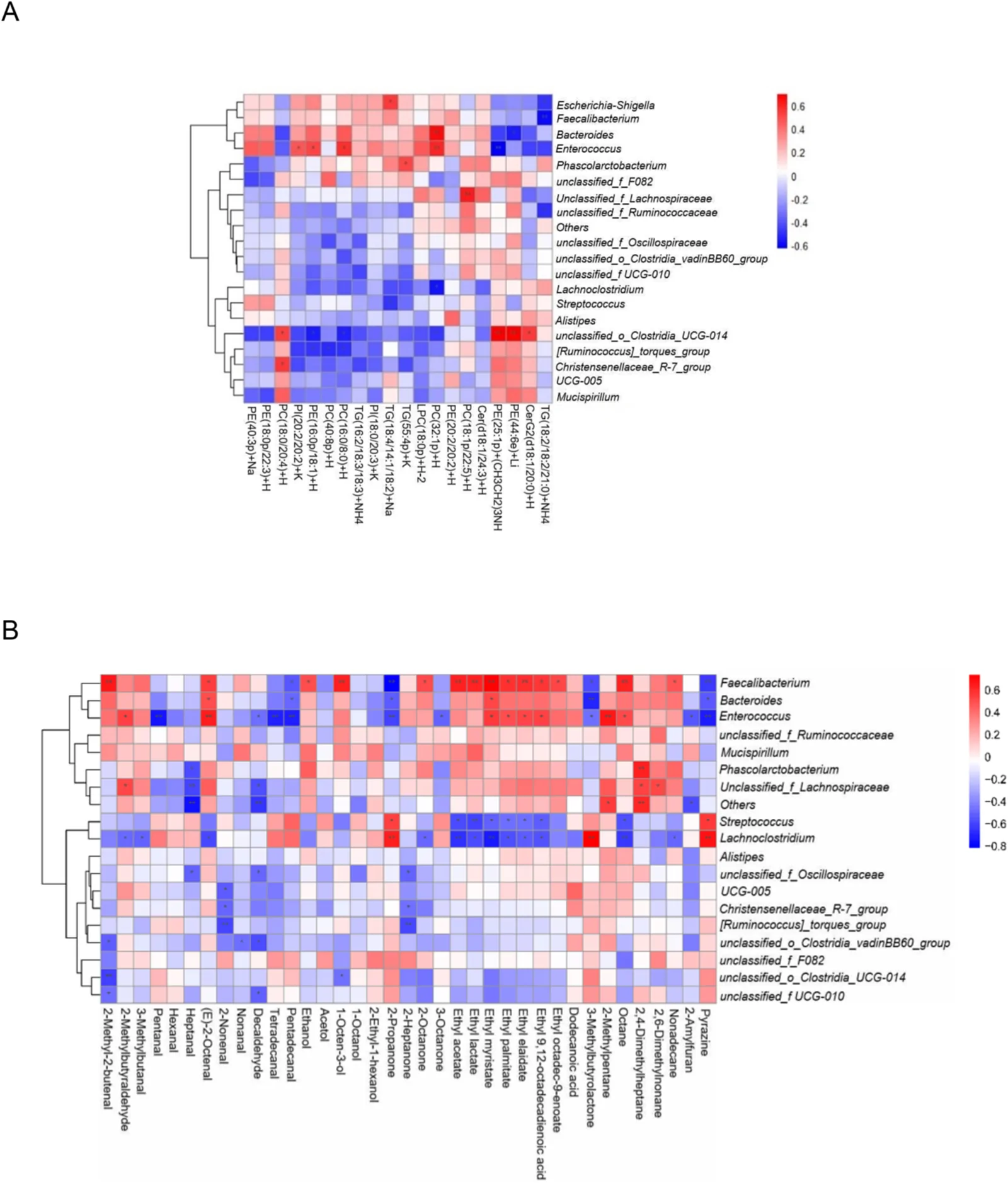
Correlations between breast muscle metabolites, volatile organic compounds (VOC), and microbial communities. (A) Correlation heatmap between gut microbial communities (top 20 genera by relative abundance) and the top 20 significantly differential lipid metabolites in breast muscle. (B) Correlation heatmap between gut microbial communities (top 20 genera by relative abundance) and breast muscle VOC (variable importance in projection [VIP] >1). ∗ Indicates a statistically significant difference between groups at *P* < 0.05; ∗∗ indicates a higher statistically significant difference between groups at *P* < 0.01. PE = phosphatidylethanolamines; PC = phosphatidylcholines; PI = phosphatidylinositols; TG = triglycerides; LPC = lysophosphatidylcholines; Cer = ceramide; CerG2 = diglycosylceramide.

## 4 Discussion

Numerous studies have shown that dietary TAU supplementation enhances ADG, decreases F/G and improves growth performance of broiler chickens (Han et al., 2020; Sandoghdar et al., 2024; Xu et al., 2020). These findings are consistent with the present results, which showed that dietary supplementation with 2.00 g/kg TAU increased the ADFI, ADG, and 56 d body weight throughout the entire rearing period. However, it has been reported that supplementing 4.00 g/kg TAU for 7, 14, and 21 d in the diet of broilers aged 21 d or increasing TAU to 8.00 g/kg from hatching to 42 d had no impact on ADG, ADFI, and feed conversion ratio (Huang et al., 2014). Research revealed that replacing 15%, 30%, and 45% methionine in the diet with TAU did not significantly alter the growth performance of the chicks aged 1 to 42 d (Ghashghaei et al., 2025). These previous studies indicate that TAU concentration and duration of supplementation are the main factors for determining whether birds respond to dietary TAU supplementation, in addition to breed and experimental conditions. A previous study has shown that TAU can stimulate the release of appetite-related hormones and effectively promote weight gain in broilers (He et al., 2019). As an amino acid, TAU promotes protein synthesis and proliferation of muscle cells through the phosphoinositide 3-kinase and mechanistic target of rapamycin (PI3K-mTOR) pathway (Hao et al., 2022). However, this study is the first to employ a local chicken breed and has the longest experimental period compared with previous studies, which may help explain the present findings. Further research is needed to explore the molecular mechanisms by which TAU regulates growth.

Similar to previous reports in broiler chickens, increasing TAU level from 0.125 to 8.00 g/kg did not affect carcass, leg, and breast percentage relative weights or meat quality including pH at 45 min and 24 h, meat color, shear force, and drip loss (Huang et al., 2014). Dietary supplementation with 2.00 g/kg TAU decreased abdominal fat rate by reducing fatty acid synthesis and enhancing lipolysis through upregulation of hormone-sensitive lipase and carnitine palmitoyltransferase 1 (Han et al., 2020; Kim and Park, 2002)). Broilers fed a diet supplemented with TAU at a level of 5.00 g/kg for only 14 d did not change pH _45 min_, but led to higher pH _24 h_ and lower drip loss percentage in breast muscle (Xu et al., 2020). The improved water-holding capacity may be attributed to TAU's ability to enhance the body's antioxidant status through direct and indirect pathways. Studies have revealed that TAU regulates the production of free radicals by maintaining mitochondrial membrane stability rather than directly scavenging them (Surai et al., 2021). In addition, TAU has been shown to interact with various transcription factors such as Nrf2 and nuclear factor kappa B (NF-κB), activating superoxide dismutase, catalase, and glutathione peroxidase, which led to a decline lipid peroxidation and enabled muscles to maintain their fluid content (Luchkova et al., 2024; Xu et al., 2020). Indeed, in the present study, the concentration of MDA and the activity of LDH decreased, while the activities of SDH and IDH increased in breast muscle, indicating improved muscle antioxidant status. This enhanced antioxidant defense system is particularly relevant because the primary components of IMF are unsaturated fatty acids (UFA), which are highly susceptible to lipid peroxidation (Hou et al., 2023; Marchewka et al., 2023; Wang et al., 2023). Consistent with this, dietary TAU supplementation decreased C18:0 and increased MUFA in breast muscle, especially C18:1n-9 and C18:2n-6. Previous research has shown that a diet containing a moderate level of MUFA and PUFA enhanced the quality and flavor of poultry meat (Cui et al., 2024). Interestingly, the present study was the first data available about dietary TAU supplementation in chicken meat fatty acid profiles. This finding could be due to TAU being involved in lipid metabolism and enhancing antioxidant status in poultry (San et al., 2023; Surai et al., 2021).

Furthermore, to better elucidate the role of TAU in flavor formation, the control (CON), medium (TAU500), and high (TAU2000) supplementation groups was selected for multi-omics analysis. Lipid components in the breast muscle were analyzed using LC-MS-based lipidomics, which identified major lipid classes, such as TG, DG, PE, and PC. Among the top 20 differential metabolites, PE, TG, PC, PI, LPC, Cer, and CerG2 were predominant. The favorable taste of breast muscle in meat-type birds was related to the presence of TG, PE, and PC (Cao et al., 2024; Chen et al., 2024). While PE has a unique role in regulating chicken breast meat taste via the formation of aroma (Zhang et al., 2024). This study found that dietary 2.00 g/kg TAU supplementation markedly increased the levels of 4 PE, 2 PC, 3 TG, 2 PI, and 1 LPC in breast muscle, which indicated that TAU has a certain effect in improving meat flavor. The IMF is recognized as a crucial factor affecting meat quality, which directly influences the tenderness, water-holding capacity, and flavor (Cao et al., 2023; Song et al., 2025). The IMF primarily consists of TG, PC, PE, phospholipids and structural lipids, which can improve muscle tenderness and flavor (Hou et al., 2023; Marchewka et al., 2023). Furthermore, a study has revealed that 4 major lipid classes were identified in the IMF of chicken breast muscle, of which were PC, PE, PG, and PI (Wang et al., 2023). These lipid substances are known to correlate with meat flavor precursors in poultry (Cao et al., 2024; Chen et al., 2024), suggesting a potential link between TAU-induced lipid changes and flavor development. Furthermore, the increasing IMF content by carbohydrate response element-binding protein (ChREBP) activation in chickens tended to improve the flavor profile of the meat (Wang et al., 2024). However, direct sensory evaluation is required to confirm this relationship. It is known that TG in circulation originates from endogenous synthesis pathways (including *de novo* lipogenesis) or dietary lipid processing. Their regulation is modulated by the transcriptional activity of lipid metabolism mediators, notably *PPAR* isoforms, *FASN*, *SREBP1*, *LPL*, *SCD*, *ELOVL* fatty acid elongases, *FABP* transporters, *ACSL5*, and the fatty acid uptake regulator *SLC27A1* (Cui et al., 2024; Liu et al., 2019; Qiu et al., 2017; Song et al., 2025). The *PPARα* expression shows significant positive associations with IMF in both pectoralis major and thigh muscles (Qiu et al., 2017). Activated *PPARα* stimulates expression of lipid metabolic enzymes, including *CPT1* (fatty acid transport) and *ACOX1* (peroxisomal β-oxidation), enhancing both fatty acid synthesis and catabolism. The process contributes significantly to the increase in IMF within muscle tissues (Chen et al., 2022). In this study, dietary TAU increased the expression levels of *PPARα*, *ACACA*, and *SREBP1,* while decreasing those of *ACOX1*, *CPT1A*, and *ACSL5*, which proved that TAU supplementation reduced lipid catabolism, rather than increased lipid anabolism, to some extent and enhanced IMF deposition. As IMF accumulation reflects the balance between lipid synthesis and degradation, suppressed fatty acid *β*-oxidation (indicated by downregulated *CPT1A* and *ACOX1*) shifts fatty acids toward TG storage, thereby promoting IMF deposition. More research is required to understand how TAU in chicken diets reduces fatty acid oxidation and increases muscle IMF deposition.

Taurine has been shown to have an impact on the intestinal environment; the relationship between TAU and intestinal microbes has attracted much attention in recent studies. Studies have shown that the gut microbiota play a role in enhancing the flavor and quality of meat (Li et al., 2022; Liu et al., 2016; Xu et al., 2024). The current research found that TAU increased the genera *Bacteroides*, *Faecalibacterium* and *Enterococcus* and decreased the genera *Streptococcus* and *Lachnoclostridium*. *Bacteroides*, which are highly colonized in distal parts of intestine, are essential for complex carbohydrate degradation and short chain fatty acids (SCFA) production (Fan et al., 2023). *Faecalibacterium* displays a major role in gut homeostasis that produces butyrate as a major energy source for enterocytes (Zhou et al., 2018). *Enterococci* have been demonstrated to enhance the activities of antioxidative enzymes through microbial–host interactions (Yu et al., 2019). *Streptococcus*, known as an opportunistic pathogenic bacterium, was enriched in association with respiratory disease (Enaud et al., 2020). Hence, TAU had a role in increasing the number of beneficial bacteria and reducing the number of intestinal pathogenic bacteria. Further evidence has shown that meat flavor and aroma are influenced by altering the cecal microbial composition and regulating the metabolic pathway of fatty acids (Liu et al., 2023a, Liu et al., 2023b; Ruan et al., 2024; Xu et al., 2024). Research indicates that dietary supplementation with *Clostridium butyricum* promotes the generation of sulfur-containing compounds, aldehydes, alcohols, ketones, and furans within avian pectoral muscle (Liu et al., 2018). This finding underscores the potential impact of *Clostridium butyricum* on the biochemical composition of poultry products. Chickens fed with *Pediococcus pentosaceus* significantly increased the contents of (E)-2-heptenal, (E, E)-2,4-nonadienal, and certain C6–C9 UFA in the breast meat (Wang et al., 2017). Diet supplemented with *Brevibacillus laterosporus* improved the flavor of chicken breast meat by increasing 1-octen-3-ol, hexanal, and octanal (Liu et al., 2023a, Liu et al., 2023b). Ducks fed lactic acid bacteria significantly enhanced meat flavor through increasing pentanal, hexanal, heptanal, 1-octen-3-ol, 2,3-octanedione, and 2-pentylfuran (Xu et al., 2024). In the present study, *Faecalibacterium* and *Enterococcus* had a positive correlation with some aldehydes and esters, such as (E)-2-octenal, ethyl elaidate, ethyl palmitate, etc. *Streptococcus* and *Lachnoclostridium* had negative correlations with esters, such as ethyl acetate, ethyl elaidate, ethyl lactate, etc. These results suggested that chickens fed diets supplemented with TAU may influence meat flavor by regulating the gut microbial community in chickens. However, it should be emphasized that these findings only demonstrate correlation, not causation. The causal link between these flavor substances and the gut microbiome needs further clarification.

## 5 Conclusion

The present results demonstrated that dietary TAU supplementation at 2.00 g/kg improved specific parameters of meat quality in yellow-feathered broiler chickens, particularly by promoting IMF deposition and modulating the profile of VOC in the breast muscle. Moreover, TAU influenced the gut microbiota structure, notably increasing the relative abundance of *Faecalibacterium* and *Enterococcus*, which may help to improve the flavor profile of the meat. Further mechanistic research is required to clarify the precise role of these bacteria in VOC formation and to confirm the potential of TAU as a nutritional additive for improving meat quality in commercial settings.

## Credit Author Statement

**Yujie Huang:** Writing – original draft, Investigation, Formal analysis, Data curation. **Ahmed Mohamed Fouad:** Writing – review & editing, Methodology, Conceptualization. **Jinling Ye:** Validation, Methodology, Data curation. **HebatAllah Kasem El-Senousey:** Validation, Methodology, Data curation. **Xiajing Lin:** Validation, Methodology, Data curation. **Sai Zhang:** Validation, Methodology, Data curation. **Yongwen Zhu:** Supervision. **Shijuan Yan:** Supervision. **Zongyong Jiang:** Supervision. **Shouqun Jiang:** Supervision. **Dong Ruan:** Writing – review & editing, Methodology, Funding acquisition, Conceptualization.

## Declaration of competing interest

We declare that we have no financial and personal relationships with other people or organizations that can inappropriately influence our work, and there is no professional or other personal interest of any nature or kind in any product, service and/or company that could be construed as influencing the content of this paper.
