# Supplementary material for: Microbiome-metabolomics analysis provides insight into the effects of dietary taurine on meat quality and flavor in yellow-feathered chickens (Gallus gallus)
Source: Anim Nutr. 2026 Sep 7;27:509–23. doi: 10.1016/j.aninu.2026.04.013 (PMC13757506; doi:10.1016/j.aninu.2026.04.013)
Supplement: Supplementary file 1 [file mmc1.docx]

Abbreviations and full names of genes.

| Abbreviations | Full names |
| --- | --- |
| *ACACA* | Acetyl-CoA carboxylase alpha |
| *ACOX1* | Acyl-CoA oxidase 1 |
| *ACSL5* | Acyl-CoA synthetase long-chain family member 5 |
| *CPT1A* | Carnitine palmitoyl-transferase 1 |
| *ELOVL* | **Elongation of very long-chain fatty acids**‌ |
| *FABP3* | Fatty acid binding protein 3 |
| *FASN* | Fatty acid synthase |
| *LPL* | Lipoprotein lipase |
| *PPARα* | Peroxisome proliferator activated receptor alpha |
| *SCD* | **Stearoyl-CoA desaturase**‌ |
| *SLC27A1* | Solute carrier family 27 member 1 |
| *SREBP1* | Sterol regulatory element binding protein 1 |

Table S1

Primer sequences used for quantitative real-time PCR.

| Genes | Direction | Primer sequence (5′-3′) | NCBI references | Amplicon, bp | Annealing temperature, ℃ |
| --- | --- | --- | --- | --- | --- |
| *PPARα* | Forward  Reverse | ACGAATGCCAAGGTCTGAGA  TGCAAGGATGACTCTGGCTT | NM_001001464.1 | 169 | 60 |
| *ACACA* | Forward  Reverse | ATGGGTGGAAGAGTGCGTAT  ATGGGTGGAAGAGTGCGTAT | NM_205505.2 | 157 | 58 |
| *CPT1A* | Forward  Reverse | GGCTCTGGCAGGAGCTACA  AGTAGGTCAGGACACGCTCA | XM_040700878.1 | 108 | 60 |
| *FABP3* | Forward  Reverse | GAGTACATGAAGGCGTTGGG  ACCAGCTTGCCTCCATCTAG | NM_001030889.2 | 224 | 60 |
| *FASN* | Forward  Reverse | CCTGGAGGTGCTCGATATGT  ATCAAGGAATCCAGGCCCAA | NM_205155.4 | 201 | 59 |
| *ACSL5* | Forward  Reverse | ATGGGTGGAAGAGTGCGTAT TCCAGTCTCCAGGCATTGAG | NM_001031237.3 | 157 | 57 |
| *ACOX1* | Forward  Reverse | GCACGAGGACTTGAACTTCC  GGCATGAAGAAGCGATCCTG | NM_001006205.2 | 237 | 60 |
| *SREBP1* | Forward  Reverse | CCTGGAAGAAGCATCATGGC  AGAGCACAGAGGATTCGGAG | AY029224.1 | 181 | 59 |
| β-Actin | Forward  Reverse | AAGCCAACAGAGAGAAGATGA  ACCAGAGGCATACAGGGA | NM_205518 | 101 | 60 |

Table S2

Effects of dietary taurine supplementation on carcass quality (%) of yellow-feathered chickens.

| Items | Groups | | | | | SEM | *P*-Value | | |
| --- | --- | --- | --- | --- | --- | --- | --- | --- | --- |
|  | CON | TAU250 | TAU500 | TAU1000 | TAU2000 |  | ANOVA | Linear | Quadratic |
| Slaughter rate | 89.19 | 88.93 | 88.83 | 88.70 | 88.41 | 0.447 | 0.799 | 0.846 | 0.068 |
| Semi-eviscerated rate | 81.95 | 81.96 | 81.72 | 81.41 | 81.46 | 0.563 | 0.928 | 0.969 | 0.642 |
| Fully eviscerated rate | 67.59 | 67.91 | 67.15 | 66.82 | 67.07 | 0.673 | 0.793 | 0.928 | 0.824 |
| Breast muscle rate | 12.37 | 12.50 | 12.55 | 12.57 | 12.27 | 0.283 | 0.930 | 0.745 | 0.829 |
| Thigh muscle rate | 15.24 | 15.33 | 15.40 | 15.98 | 15.83 | 0.321 | 0.384 | 0.252 | 0.663 |
| Abdominal fat rate | 5.86^a^ | 5.36^ab^ | 5.23^ab^ | 5.19^ab^ | 4.56^b^ | 0.404 | 0.030 | 0.239 | 0.027 |

CON **=** basal diet group; TAU250 = basal diet supplementation with 0.25 g/kg taurine group; TAU500 = basal diet supplementation with 0.50 g/kg taurine group; TAU1000 = basal diet supplementation with 1.00 g/kg taurine group; TAU2000 = basal diet supplementation with 2.00 g/kg taurine group; SEM = standard error of the mean.

Means within a row with different superscript letters are significantly different (*P* < 0.05). Each value represents the mean of 6 replicates (30 chickens per replicate) per treatment.

Table S3

Effects of dietary taurine supplementation on α-diversity index of cecum microbial community in yellow-feathered chickens.

| Item | CON | TAU500 | TAU2000 | SEM | *P*-value |
| --- | --- | --- | --- | --- | --- |
| Species observed | 419.4^ab^ | 442.9^a^ | 356.2^b^ | 22.81 | 0.031 |
| Shannon | 3.16^ab^ | 3.94^a^ | 3.38^b^ | 0.196 | 0.151 |
| Simpson | 0.10 | 0.08 | 0.14 | 0.029 | 0.369 |
| Chao | 531.9^a^ | 540.9^a^ | 444.8^b^ | 24.66 | 0.017 |
| Ace | 532.1^a^ | 543.5^a^ | 449.9^b^ | 23.73 | 0.017 |
| Good’s coverage | 0.990^b^ | 0.991^b^ | 0.993^a^ | 0.0023 | 0.032 |

CON **=** basal diet group; TAU500 = basal diet supplementation with 0.50 g/kg taurine group; TAU2000 = basal diet supplementation with 2.00 g/kg taurine group; SEM = standard error of the mean.

Means within a row with different superscript letters are significantly different (*P* < 0.05). Each value represents the mean of 6 replicates (30 chickens per replicate) per treatment.

A

|  |  |
| --- | --- |
| cer | 31 |
| cer g1 | 10 |
| cer g2 | 5 |
| cer g3 | 2 |
| DG | 221 |
| GM3 | 3 |
| LPC | 48 |
| LPE | 14 |
| LPG | 5 |
| LPS | 5 |
| MG | 7 |
| PC | 275 |
| PE | 216 |
| PG | 9 |
| PI | 26 |
| PS | 17 |
| SM | 53 |
| TG | 545 |

B

Fig. S1. Total lipid metabolite species distribution in breast muscle of broiler chickens (*n* = 6). (A) Total lipid metabolite species distribution in breast muscle of broiler chickens (*n* = 6). (B) Significantly different numbers of lipid metabolites in different subclasses. TG = triglycerides; PE = phosphatidyl ethanolamine; PC = phosphatidylcholine; DG = diacylglycerol; Cer = ceramide; SM = sphingomyelin; LPC = lysophosphatidylcholine; PI = phosphatidylinositol; PG = phosphatidylglycerol; CerG2 = diglycosylceramide; MG = monoglyceride; PS = phosphatidylserine; CerG3 = triglycosylceramide; GM3 = ganglioside m3; LPG = lysophosphatidylglycerol; LPS = lysophosphatidylserine; LPE = lysophosphatidylethanolamine.
